# Bionomics of *Phlebotomus argentipes* in villages in Bihar, India with insights into efficacy of IRS-based control measures

**DOI:** 10.1371/journal.pntd.0006168

**Published:** 2018-01-11

**Authors:** David M. Poché, Rajesh B. Garlapati, Shanta Mukherjee, Zaria Torres-Poché, Epco Hasker, Tahfizur Rahman, Aakanksha Bharti, Vishnu P. Tripathi, Suman Prakash, Rahul Chaubey, Richard M. Poché

**Affiliations:** 1 Department of Vector Ecology, Genesis Laboratories, Inc., Wellington, United States of America; 2 Department of Entomology, Genesis Laboratories India Private Limited, Patna, India; 3 Department of Public Health, Institute of Tropical Medicine, Antwerp, Belgium; National Institutes of Health, UNITED STATES

## Abstract

**Background:**

Visceral leishmaniasis (VL) is a deadly vector-borne disease. Approximately 90% of Indian VL cases occur in Bihar, where the sand fly, *Phlebotomus argentipes*, is the principal vector. Sand fly control in Bihar consists of indoor residual spraying (IRS), the practice of spraying the inner walls of village dwellings with insecticides. Prior researchers have evaluated success of IRS-control by estimating vector abundance in village houses, but the number of sampling periods (*n* = 2–3) were minimal, and outdoor-resting *P*. *argentipes* were neglected. We describe a large-scale field study, performed in 24 villages within two Bihari districts, during which *P*. *argentipes* were collected biweekly over 47-weeks, in cattle enclosures, houses, and outdoors in peri-domestic vegetation. The objectives of this study were to provide updated *P*. *argentipes* ecological field data, and determine if program-initiated IRS-treatment had led to noticeable differences in vector abundance.

**Principal findings:**

*P*. *argentipes* (*n* = 126,901) relative abundance was greatest during the summer months (June-August) when minimum temperatures were highest. *P*. *argentipes* were most frequently collected from cattle enclosures (~46% total; ~56% blood fed). Many sand flies were found to have taken blood from multiple sources, with ~81% having blood fed on humans and ~60% blood feeding on bovines. Nonparametric statistical tests were determined most appropriate for evaluating IRS-treatment. Differences in *P*. *argentipes* abundance in houses, cattle enclosures and vegetation were detected between IRS-treated and untreated villages in only ~9% of evaluation periods occurring during the peak period of human-vector exposure (June-August) and in ~8% of the total observations. No significant differences were detected between the numbers of *P*. *argentipes* collected in vegetation close to the experimental villages.

**Conclusion:**

The results of this study provide updated data regarding *P*. *argentipes* seasonal abundance, spatial distribution, and host preferances, and suggest vector abundance has not significantly declined in IRS-treated villages. We suggest that IRS be supplemented with vector control strategies targeting exophagic, exophilic *P*. *argentipes*, and that disease surveillance be accompanied by rigorous vector population monitoring.

## Author summary

Visceral leishmaniasis is a disease caused by a deadly vector-borne parasite (*Leishmania donovani*) transmitted to man by phlebotomine sand flies. Indoor residual spraying (IRS), performed within village dwellings, is the primary means of sand fly control performed in Bihar, India and more explicit methods of evaluating the success of control are warranted. A field-based study was conducted to collect ecological sand fly data for use in evaluating the effectiveness of IRS in reducing relative sand fly abundance. Results indicate that sand flies blood feed primarily on humans and cattle and are most frequently found within cattle enclosures. Results further suggest IRS-treatment has a limited impact on vector density. Our approach incorporates detailed evaluation of sand fly spatial distribution (cattle enclosures, houses, vegetation), seasonal fluctuations in abundance, host blood meal preferences within Bihari villages, and dates of IRS performed within treated villages. Hence, this study provides an explicit means of monitoring vector populations and evaluating control measures in Bihar.

## Introduction

Visceral leishmaniasis (VL), also known as kala-azar, is a parasitic disease transmitted by infected female phlebotomine sand flies. VL is fatal if left untreated, and annually there are an estimated 500,000 human VL cases and 50,000 deaths worldwide, making it the second deadliest parasitic disease surpassed only by malaria [1–2]. The Indian subcontinent accounts for approximately 67% of the reported human cases of VL, the majority of which occur in areas of extreme poverty [3]. Approximately 90% of cases in India occur in Bihar [4]. The known vector of VL in the Indian subcontinent is the sand fly species *Phlebotomus argentipes* [5–6] which transmits the pathogen (*Leishmania donovani*) anthroponotically with no known animal reservoir [7]. In 2005, the World Health Organization (WHO) helped the governments of Bangladesh, India, and Nepal to launch a regional elimination initiative to reduce VL cases in the Indian subcontinent to less than one case per 10,000 people in endemic districts by 2015, two of the main strategies being: integrated vector management and effective disease and vector surveillance [8]. Although attempts have been made to control phlebotomine sand fly populations, organization and implementation of effective vector control programs have been hindered largely because of uncertainties surrounding phlebotomine sand fly ecology [9].

### Vector ecology

On the Indian subcontinent, researchers have made attempts to answer questions regarding aspects of *P*. *argentipes* field ecology, including: seasonality, spatial distribution, and host preference. Researchers have suggested that the abundance of *P*. *argentipes* is associated with ecological parameters such as temperature and precipitation [10]. Results of a more recent 12-month study conducted in three Bihari villages in the Saran district, during which 52,653 sand flies were trapped using light traps, suggest *P*. *argentipes* numbers are typically highest during the months when evening temperatures are warmest and precipitation is increased (June, July, August) and lowest during the coolest months (January, February, December) [11]. These results further suggested spatial distribution of *P*. *argentipes* was not limited to the inside of village dwellings, but also included outlying village vegetation. *P*. *argentipes* females are regarded as anautogenous, needing a blood meal to produce each batch of eggs [12], and results of several studies suggest they blood feed almost exclusively on bovines (cattle and domestic buffalo) and humans within rural villages [13–17].

### Vector control

Because *P*. *argentipes* is believed to be endophilic and endophagic [18], the vector control approach utilized for decades in Bihar has been indoor residual spraying (IRS), a method of applying insecticide to the inner walls of village houses and cattle dwellings. Explicit data addressing the impact of IRS on *P*. *argentipes* is limited [19–21] and, therefore, the impact of IRS on field populations is largely unknown.

A cluster randomized trial was conducted by [22] in which IRS was performed in village dwellings in India (DDT), Nepal (alpha-cypermethrin), and Bangladesh (deltamethrin), during which pre-treatment light trap collections, performed over two consecutive nights in November, were compared with post-treatment light trap collections performed over two consecutive nights in April of the following year. One issue with this study design is the lengthy period (~5-months) between IRS treatment and sample collection, during which multiple factors could influence sand fly abundance. The authors mention other caveats as being small sample sizes within sites, limiting the reliability of site-specific analysis, and the fact that the study was conducted under controlled conditions not easily applicable in a national vector control program.

Given the latter limitation, other researchers have collected *P*. *argentipes* from treated and untreated households during program-initiated DDT spraying in India [23–26]. While the results of these IRS-evaluation studies are interesting and useful, we would argue that they all share two limitations 1) a minimal number of post-IRS *P*. *argentipes* collection periods and 2) a universal neglect of outdoor sand fly population sampling. Post-treatment *P*. *argentipes* collections during the previously mentioned studies were typically conducted at ~1-month and ~5-6-months post-treatment. *P*. *argentipes* have been collected in large numbers from peri-domestic vegetation [11], and villagers sleep outdoors during warmer months [8] when vector abundance and biting rates are high [27]. Hence the exophilic, exophagic sand fly population should be monitored. Considering the sensitivity of vector abundance to environmental factors such as temperature and precipitation and the tendency *P*. *argentipes* to be captured in light traps positioned in vegetation and cattle enclosures [11,28–29], we suggest the current IRS-treatment programs could be better evaluated if *P*. *argentipes* were collected at greater frequencies and if trap locations were diversified to include cattle enclosures and outdoor locations such as peri-domestic vegetation.

### Objectives

In this paper, we describe a large-scale, 11-month field study, conducted in 24 villages in two VL-endemic districts in Bihar, India, in which sand flies were collected using United States Centers for Disease Control and Prevention (CDC) light traps (Bioquip Products, Rancho Dominguez, CA, USA; John W. Hock Company, Gainesville, FL, USA) during 24 collection periods conducted over 47-weeks. The objectives of this study were to 1) provide updated *P*. *argentipes* ecological data within villages in two Bihari districts; and 2) determine if relative *P*. *argentipes* abundance in IRS-treated villages was reduced when compared with untreated villages. By utilizing methods previously described by [11] and [17], but using a much larger sample size, field-collected *P*. *argentipes* were used to estimate: spatial distribution (cattle enclosures, houses, vegetation), temporal fluctuations in relative abundance, and host preferences of blood fed females. Additionally, we used IRS data for the study villages to compare relative *P*. *argentipes* abundance in IRS-treated and untreated villages during the 2016 season. The results of this study will provide 1) useful, current ecological information regarding *P*. *argentipes* seasonal abundance, spatial distribution, and host preference in two VL-endemic districts; and 2) an alternative means of evaluating IRS-treatment through biweekly vector monitoring, helping to determine whether integrated methods of vector management should be recommended in Bihar.

## Materials and methods

### Study area

The study was conducted in the Saran and Muzaffarpur districts of Bihar, India. Saran and Muzaffarpur are adjacent districts located ~30 km northwest (25.8560° N, 84.8568° E) ~60 km north (26.121736° N, 85.373700° E) of Patna, respectively. Summers are warm with maximum temperatures often ranging from 35–40°C. The winter months (December-February) are typically much cooler [11]. The rainy season typically occurs from July-September and April is generally the driest month.

**Fig 1:**
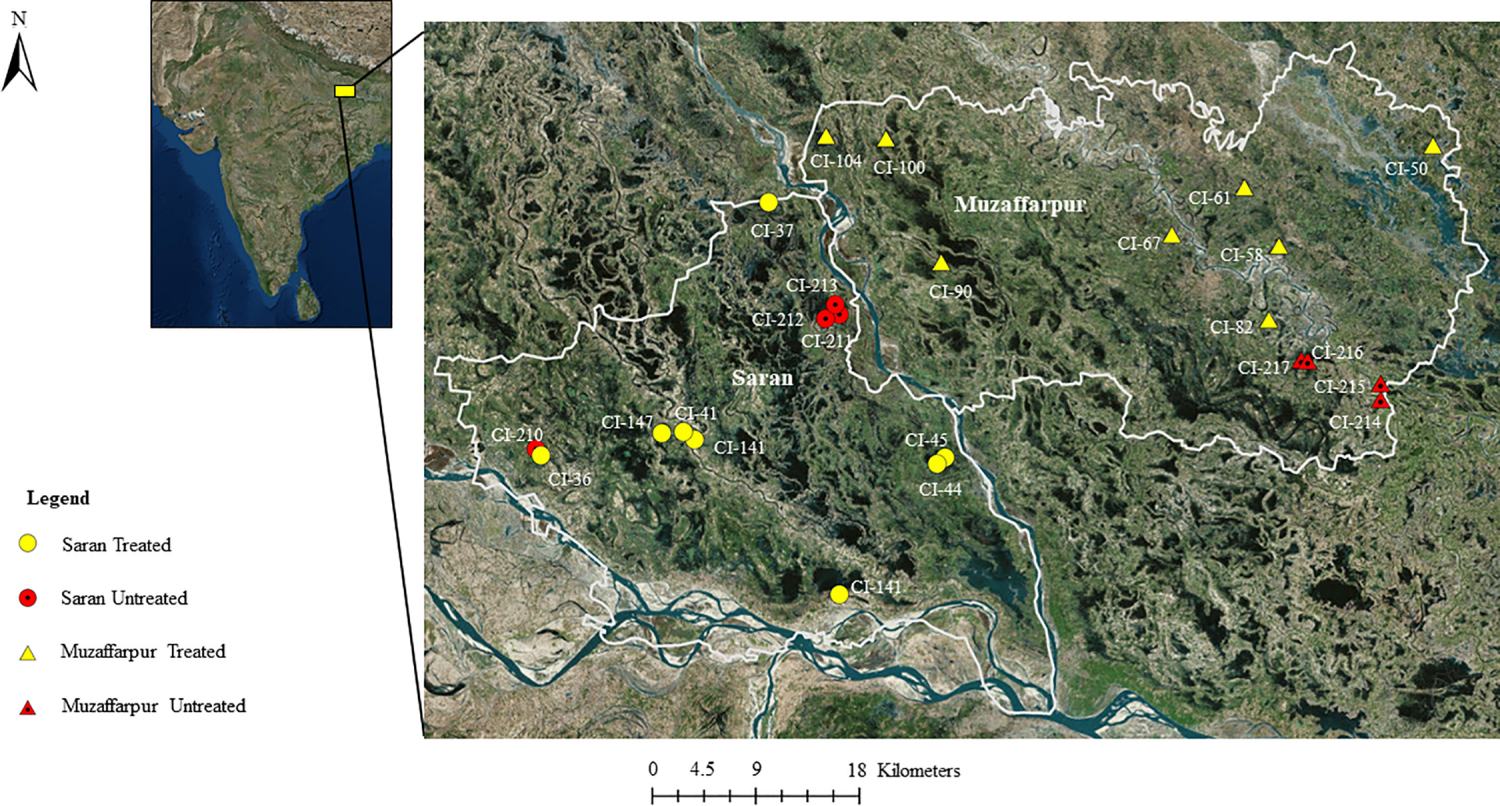
Map of 24 study villages in two VL-endemic districts in Bihar, India. Map was generated in ArcGISusing ArcMap with a World Imagery base layer (Sources: Esri, DigitalGlobe, GeoEye, i-cubed, USDA FSA, USGS, AEX, Getmapping, Aerogrid, IGN, IGP, swisstopo, and the GIS User Community).

All villages were part of a large-scale VL-incidence survey performed in 60 villages within each district (*n* = 120) in 2015. At the beginning of 2016, 12 villages in each district were selected for sand fly collection (*n* = 24) (Fig 1) with the aim of collecting sand flies from February-December. Prior to study initiation, it was discovered that two rounds of IRS application had been performed within several villages in both districts in 2015 and that application would be repeated in 2016. To evaluate the impact of IRS on vector abundance, we selected 8 IRS-treated villages and 4 untreated villages within each district (*n* = 16; *n* = 8) for CDC light trap collection. The main criteria for village selection were a population of >1,000 villagers, confirmed cases of VL spanning 2013–2015, and IRS-status (treated or untreated). All villages shared similar bioclimatic and agricultural characteristics. Livestock ownership was common with cows (*Bos taurus*, *Bos indicus*), domestic buffalo (*Bubalus bubalus*), and domestic goats (*Capra aegagrus hircus*) prevalent in each village. Dwellings were constructed primarily of thatch, mud, brick, and/or concrete, consisting mainly of human houses and cattle enclosures (cattle sheds, houses cohabitated by humans and bovine). At night, livestock were typically tethered to stakes, and were kept in rooms within cattle enclosures or outdoors adjacent to human dwellings and/or cattle enclosures.

Local weather data including daily temperature (°C), relative humidity (%), and precipitation (mm) (February 10-December 31, 2016) were collected from the closest, attainable weather monitoring station, located at Jay Prakash Narayan International Airport (VEPT) in Patna [30].

### Sand fly collection

CDC light traps were used to collect sand flies in study villages. Twelve (12) CDC light traps were set, in fixed locations, within each of the 24 study villages (*n* = 288 trap-nights per collection week) biweekly from February 10-December 29, 2016 (24 collection periods). During each collection period, CDC light trapping was performed over two consecutive nights with 12 villages being sampled on night-1 and the remainder on night-2. Collection was not conducted in January because adult sand flies are typically not active during this period [11]. CDC traps were set in randomly selected homes (*n* = 4), cattle enclosures (cattle sheds or dwellings cohabitated by bovines and humans) (*n* = 4), and outdoors in peri-domestic village vegetation (*n* = 4) to better estimate *P*. *argentipes* spatial distribution. Traps were identified by a unique code and waypoints taken with a handheld GPS (Garmin Etrex 30, Olathe, KS, USA). Traps were positioned with the fan ~1 m above the ground [11]. In peri-domestic vegetation, CDC traps were fitted with protective lids to shield the mechanical components from falling debris or rain. Traps were set at ~18:00 and removed at ~06:00 the following morning. Trap catches were individually numbered, transported to the laboratory in Patna, and frozen at -20°C until further processing. Because dry-ice was not available within the state of Bihar, we were unable to compare the efficiency CDC light traps with CO_2_ traps. Permission was received from village residents prior to conducting light trapping.

### Sand fly counts and identification

Individual CDC light trap catches were uniquely numbered and identified by trap location and date of capture. Captured sand flies were separated from other arthropods, counted, identified morphologically by species, sexed, and females confirmed as unfed, blood fed, or gravid. If sand fly species was uncertain, specimens were placed under a dissecting microscope and identified morphologically by observing the male genitalia and the female spermatheca, the latter which required dissection [31].

Molecular species confirmation of sand flies was performed routinely on a random sample of specimens from each village. Whole sand fly DNA was extracted individually according to manufacturing instructions using the DNAzol reagent (ThermoFisher Scientific, Waltham, MA, USA). Individual sand fly DNA was amplified using forward primer 5’ -TCG AAT CTA TGG GTG GT-3’ and reverse primer 5’- CAC AAT CCC AAC CAC GAA G-3’ for the 18S rRNA target gene. Restriction endonuclease digestion was performed on the PCR product of 18S r RNA using HAE II and HAE III enzymes. Banding pattern after Gel electrophoresis confirmed the identification of *P*. *argentipes* [32].

### Sand fly blood meal analysis

After completing counts and identification, blood fed female *P*. *argentipes* were separated and placed into dry 1.5 ml centrifuge tubes and stored at -20°C. Sand fly heads were removed from the bodies prior to analysis. DNA was then extracted from the abdomen and thorax of blood fed sand flies. The cytochrome *b* gene region (344 bp conserved mitochondria gene) was amplified using bio-tinilated universal primers designed earlier by [33]. Cow blood was used as positive control and sterile water as negative control. The amplified products were used in reverse line blotting hybridization as probes, followed by chromogenic detection. Immobilization, hybridization and detection were done according to methods of [33].

Previously developed source-specific probes for human, cow, buffalo, goat, and chicken were used to analyze the blood meal contents for the blood fed sand flies collected during this study. These techniques allowed for detecting multiple host species in a single *P*. *argentipes* blood meal. These procedures are described in detail by [17,33].

### Sand fly spatial distribution

*P*. *argentipes* successfully analyzed by cytochrome *b* amplification and reverse line blotting were categorized by blood meal content (human, bovine, goat, etc.) and trap placement (cattle enclosure, house, vegetation). A Pearson’s chi square (*X*^*2*^) test was used to estimate dependence of blood meal content on the spatial distribution of blood fed *P*. *argentipes* (trap placement) (*p* = 0.05).

*P*. *argentipes* distribution was further evaluated by comparing the relative abundance of all *P*. *argentipes* within trap placements. Differences between and within trap catches in cattle enclosures, houses, and vegetation in Muzaffarpur, Saran, and cumulative districts were assessed using Analysis of Variance (ANOVA) (*p* = 0.05) followed by Tukey’s W procedure (*p* = 0.05).

### Sand fly seasonal fluctuations

To estimate relative *P*. *argentipes* seasonal fluctuations in abundance, we compared overall *P*. *argentipes* seasonal abundance in Muzaffarpur and Saran and compared seasonal abundance in cattle enclosures, houses, and vegetation within each district. More specifically, we compared the mean *P*. *argentipes* per trap-night per month (*n* = 11) and per collection period (*n* = 24) within districts and trap placements. A nonparametric Sign test (*p* = 0.05) was used to estimate whether changes in monthly and biweekly abundance were significantly different between districts and between trap placements within districts. That is, whether *P*. *argentipes* abundance tended to increase and decrease in relative unison throughout the calendar year.

### Comparing IRS-treated with untreated villages

The specific 2016 IRS spray dates for each village were provided by CARE India (Patna, Bihar) (S1 Table), written on the walls of the village dwellings by the applicators, and were also confirmed by the home owners. The inner walls of houses and cattle enclosures in IRS-treated villages had been sprayed with two rounds of alpha-cypermethrin (5%) wettable powder (WP) at a rate of 25mg/m^2^, and the dates of application varied on a village-to-village basis. The first round of IRS application ranged from April 1–May 30 in Muzaffarpur and April 6-June 2 in Saran (S1 Table). The second round of IRS application ranged from August 20-November 14 and September 15-November 28 in Muzzaffarpur and Saran, respectively. All IRS-treated study villages received two rounds of IRS application in 2015. None of the untreated study villages had received IRS-treatment in over three years. In response to insecticide resistance, alpha-cypermethrin (a synthetic pyrethroid) has replaced DDT in 15 Bihari districts [34].

Because CDC light trap collections were performed during on-going program-initiated IRS-treatment, we were unable to collect pre-treatment (baseline) data. We first compared the mean *P*. *argentipes* per trap-night per collection period (±SE) in IRS-treated and untreated villages within each district. We were specifically interested in differences occurring during the months with the highest relative vector abundance (June-August) and, hence, whether the first round of IRS application may have influenced *P*. *argentipes* abundance during this period of peak human-vector exposure. Nonparametric methods deemed sufficient for most skewed analyses [35], were considered most appropriate to analyze differences between IRS-treated and untreated villages. Hence, a Wilcoxon rank sum test was used to estimate differences in relative abundance of *P*. *argentipes* in IRS-treated and untreated villages (*p* = 0.05) by district. One of the villages in Saran (CI-210), initially untreated during the first round in 2016, received IRS application during the second round (September 21), due to newly reported cases within the village, and therefore was excluded from the statistical analysis after September. We additionally excluded February and December from statistical analysis due to low vector abundance.

## Results

April and December on average had the high and low maximum temperatures with means of 40.6°C and 21.6°C, respectively (S2 Table). The highest mean minimum temperatures were recorded in June, July and August. July was the rainiest month, with ~202.9 mm precipitation being recorded and nearly all precipitation (>99%) was recorded May-October. Maximum humidity was generally high (>80%) with the low point being in April (59.6%). Minimum humidity ranged from 16.9% in April to 69.1% in September.

### Sand fly collection, counts and identification

During 24 collection periods, occurring over 47-weeks (6,349 trap-nights), a total of 155,908 sand flies were captured, counted, and identified (S3 Table), of which 126,901 were *P*. *argentipes* (Males = 76,904; unfed females = 44,133; blood fed females = 2,299; gravid (no blood seen) females = 3,565) (Table 1). Individual trap-night yields ranged from 0–3,248 *P*. *argentipes*. A total of 76,516 and 50,385 *P*. *argentipes* were caught in CDC light traps in villages in Muzaffarpur and Saran, respectively. Twenty-four thousand two hundred eighty (24,280) *Sergentomyia* spp. and 1,477 *P*. *papatasi* were also collected.

**Table 1:** Summary of the total number of *P*. *argentipes* collected from houses (H), cattle enclosures (CE), and peri-domestic vegetation (V) between February 10-December 29, 2016.

District	Location	*Phlebotomus argentipes*				
		M	F (UF)	F (BF)	F (G)	Total
Muzaffarpur	H	15052	10176	482	678	26388
	CE	21391	11729	634	920	34674
	V	9346	5531	176	401	15454
	Total	45789	27436	1292	1999	76516
Saran	H	7221	4137	160	434	11952
	CE	15252	7221	679	808	23960
	V	8642	5339	168	324	14473
	Total	31115	16697	1007	1566	50385

M = Male, F = Female, (UF) = Unfed, (BF) = Blood fed, (G) = Gravid

In total, 38,583 and 30,236 female sand flies were identified by dissection and PCR analysis, respectively (S1 Fig, S2 Fig). Three thousand two hundred twenty-four (3,224) female *Grassomyia indica* were also identified. Twenty six (26) females could not be identified.

### Blood meal analysis

**Fig 2:**
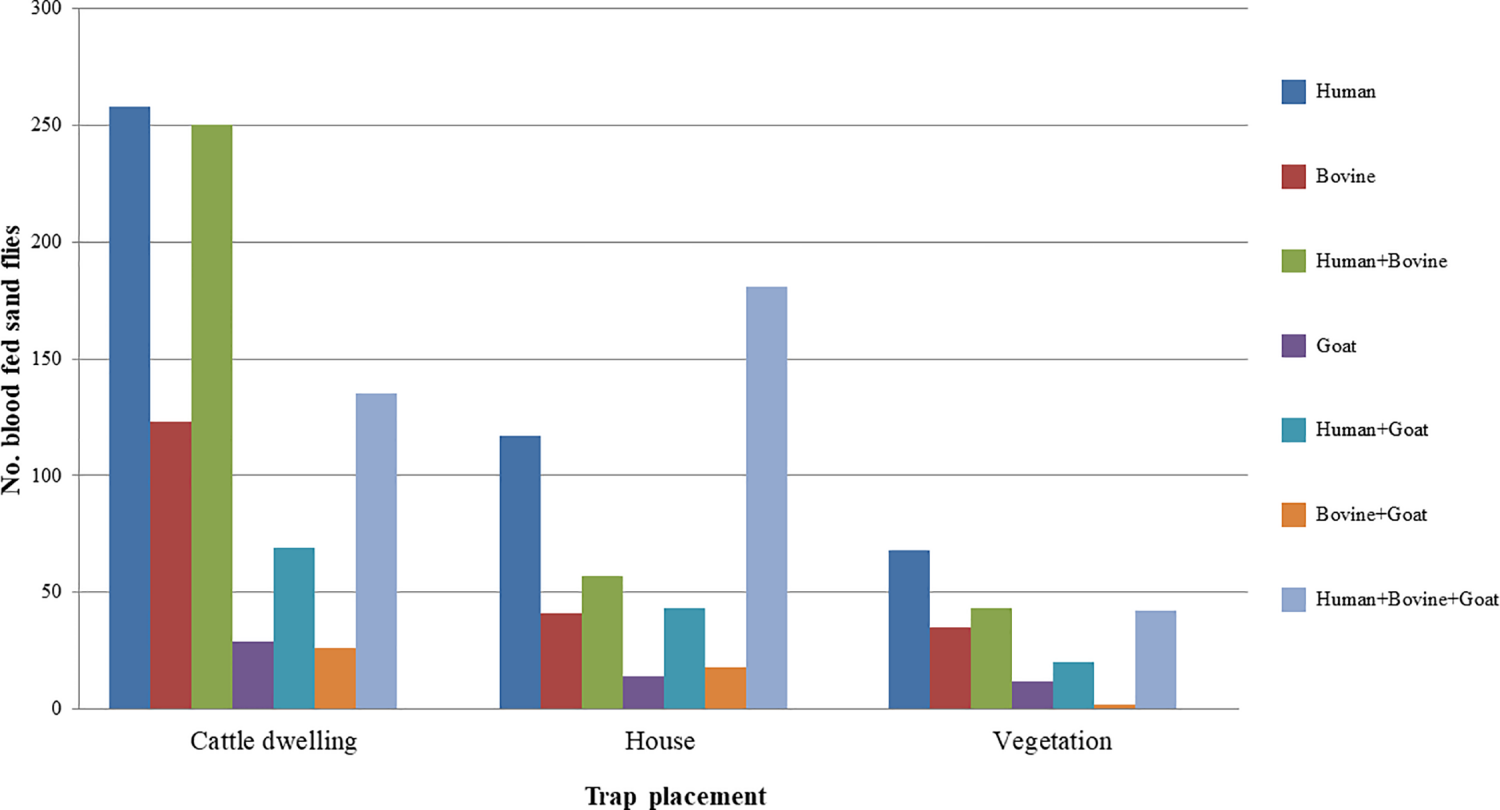
Blood meal sources and trap placement of *P*. *argentipes* successfully analyzed using reverse-line blotting.

Of the 2,299 blood fed *P*. *argentipes* collected, a total of 1,583 were successfully analyzed using *cytochrome b* PCR and reverse-line blot analysis (Fig 2). Blood meal content consisted of human, bovine, and goat blood, exclusively. Approximately 60% *P*. *argentipes* blood meals were positive for more than one host species. Of all blood fed *P*. *argentipes* successfully analyzed, the majority were positive for human (~81%) and/or bovine (~60%) blood.

### Spatial distribution

Blood meal status was dependent on trap placement (*X*^*2*^; *p*<0.0001), primarily in cattle enclosures. Overall, blood fed female *P*. *argentipes* were most frequently collected in cattle enclosures (~56%) followed by houses (~30%), and vegetation (~14%) (Fig 2). As would be expected, more *P*. *argentipes* containing bovine blood meals were collected in cattle enclosures (~56%) than in houses (~31%) or vegetation (~13%). But more *P*. *argentipes* containing human blood meals were also collected in cattle enclosures (~55%) than in houses (~31%). This greater dependence on cattle enclosures was observed in all *P*. *argentipes* blood meal content types except for mixed human, bovine and goat blood meals, which were collected primarily from houses (~50%), followed by cattle enclosures (~38%) and vegetation (~12%).

The overall *P*. *argentipes* spatial distribution was relatively similar to that of blood fed females, with more being collected from cattle enclosures (~46%) followed by houses (~30%) and outlying vegetation (~24%) (Table 1). The mean *P*. *argentipes* per trap-night were determined to be significantly different between trap placements in Muzaffarpur (ANOVA; *p*<0.0001), in Saran (ANOVA; *p* = 0.0018), and in cumulative districts (ANOVA; *p*<0.0001). Differences within trap placements were estimated between cattle enclosures and vegetation in Muzaffarpur (Tukey’s W; *p*<0.0001), in Saran (Tukey’s W; *p* = 0.0234), and in cumulative districts (Tukey’s W; *p*<0.0001); between cattle enclosures and houses in Saran (Tukeys W; *p =* 0.0020) and in cumulative districts (Tukey’s W; *p =* 0.0015); and between houses and vegetation in Muzaffarpur (Tukey’s W; *p =* 0.0397).

### Sand fly seasonal fluctuations

#### Monthly

**Fig 3:**
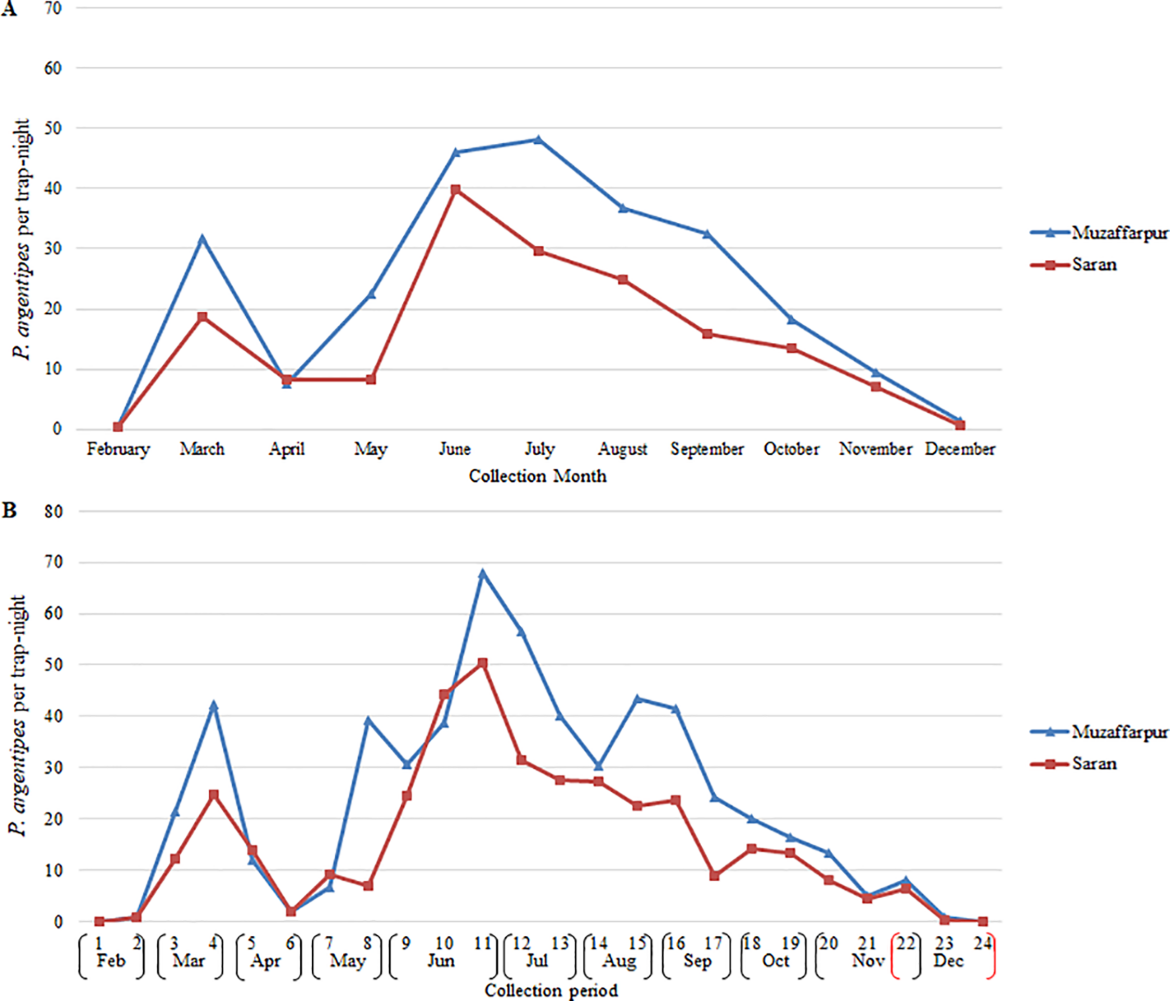
*P*. *argentipes* per trap-night per month in Muzaffarpur and Saran by a) month and b) trapping period.

Results of CDC light trapping in Muzaffarpur and Saran suggested similar fluctuations in the monthly relative abundance of adult *P*. *argentipes* (Fig 3A). The first peak in adult *P*. *argentipes* occurred in March followed by a sharp decrease in April. Relative abundance was highest during the summer months (June, July, August) and lowest in February and December. Differences in general trends in monthly relative abundance of *P*. *argentipes* in Muzaffarpur and Saran, were nearly significant (Sign test; *p* = 0.0654).

**Fig 4:**
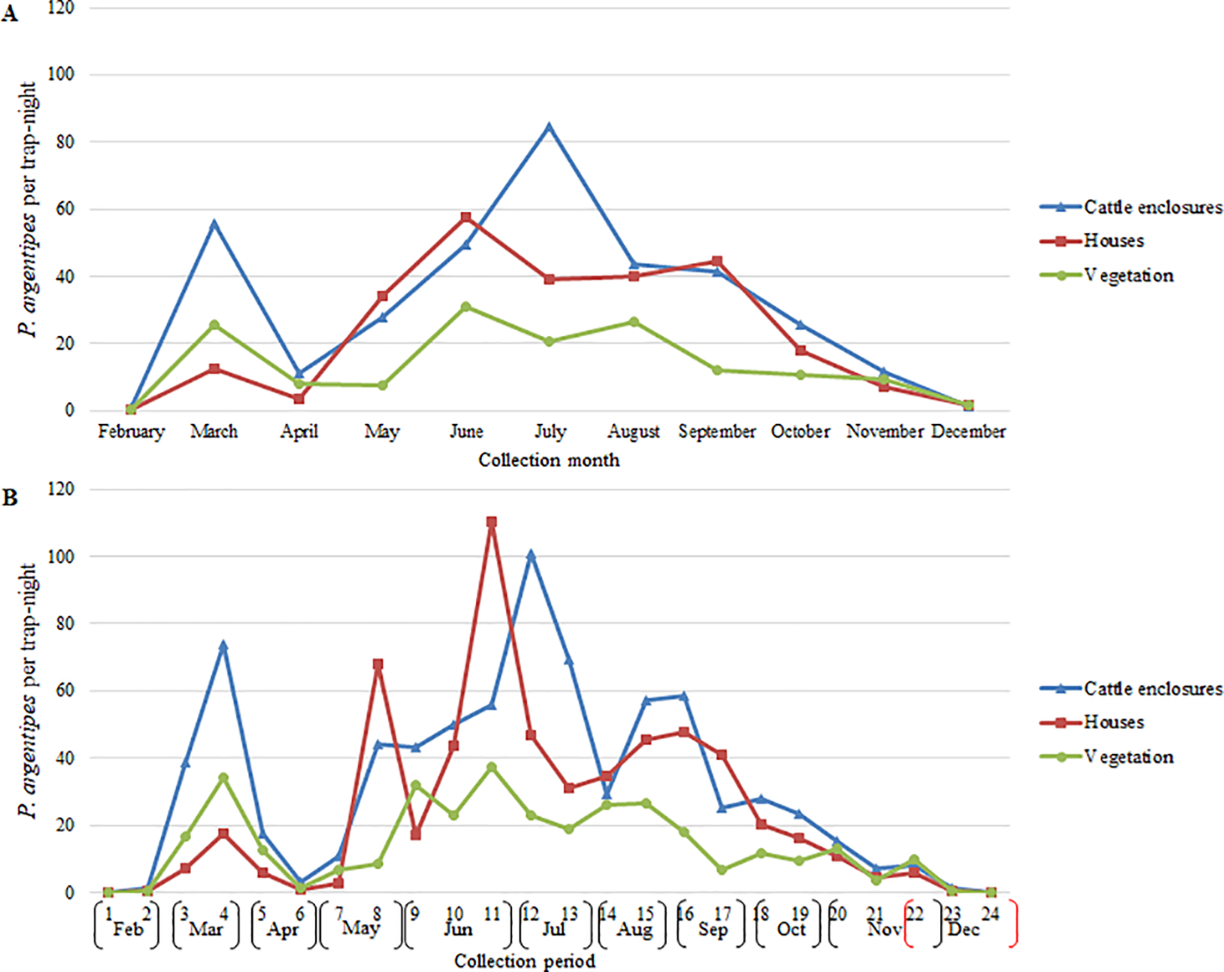
*P*. *argentipes* per trap-night in cattle enclosures, houses and vegetation in Muzaffarpur by a) month and b) trapping period.

**Fig 5:**
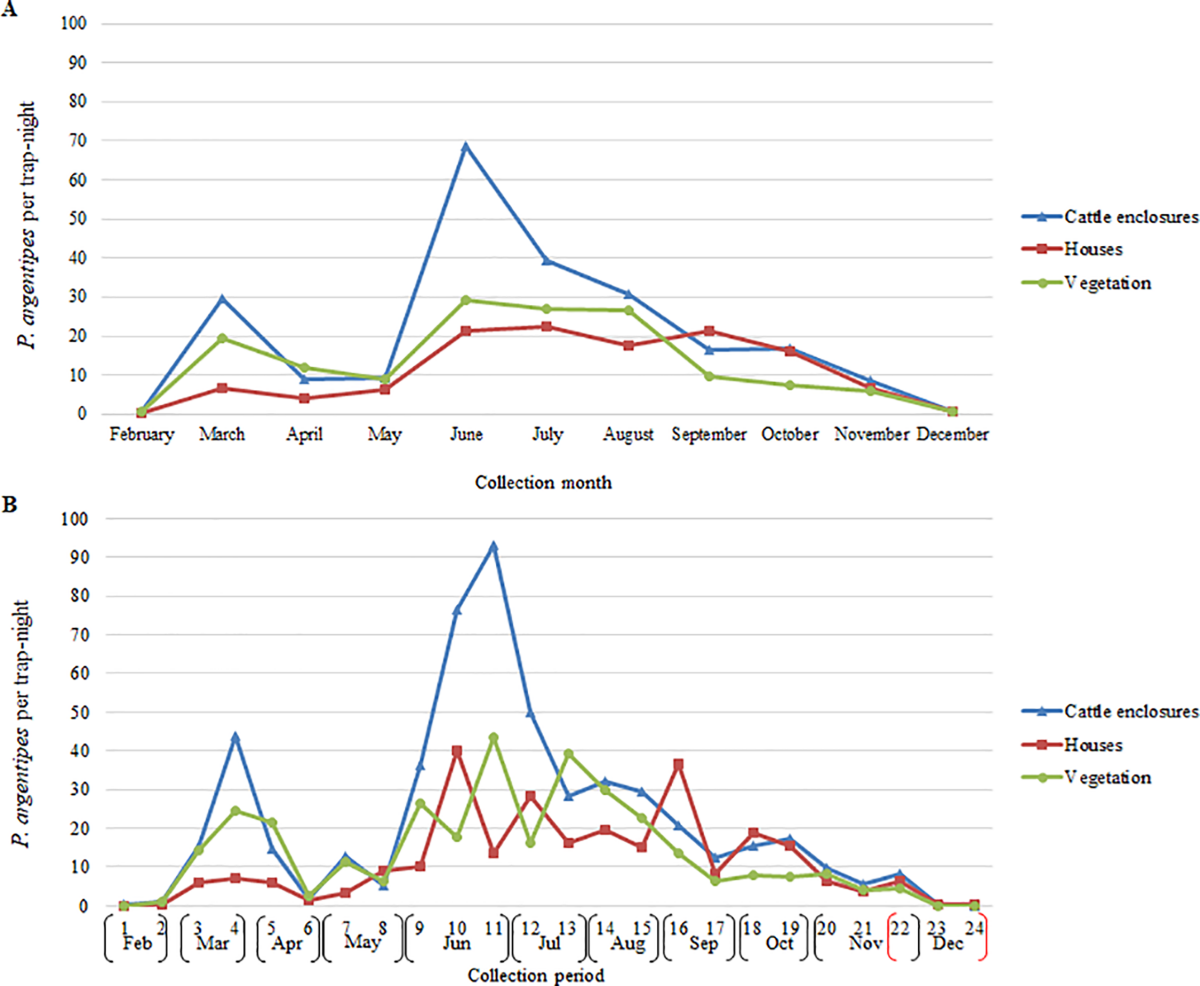
*P*. *argentipes* per trap-night in cattle enclosures, houses and vegetation in Saran by a) month and b) trapping period.

The mean number of *P*. *argentipes* per trap-night was greatest in traps positioned in cattle enclosures in July (84.7) and June (68.7) in Muzaffarpur and Saran, respectively (Figs 4A and 5A). The largest peaks in the mean *P*. *argentipes* per trap-night occurred in June in houses and vegetation in Muzaffarpur (57.4, 30.9) and Saran (22.4, 29.3). *P*. *argentipes* numbers were lowest in February and December for all trap location types in both districts. Differences in monthly fluctuations in relative abundance were most apparent when comparing cattle enclosures with vegetation (Sign test; *p* = 0.0117), and no significant differences were determined when comparing cattle enclosures to houses or vegetation to houses.

#### Biweekly

Results suggested that fluctuations in biweekly *P*. *argentipes* relative abundance were less comparable. In both districts, the first peak occurred during collection period-4 in March and the largest peak occurred during collection period-11 in June (Fig 3B). Numbers declined noticeably during collection period-6 in April. However, villages in Muzaffarpur on average experienced a greater number of *P*. *argentipes* peaks (collection periods-4,8,11,15) than villages in Saran (collection periods-4,11). General trends in biweekly relative abundance within Muzaffarpur and Saran were determined to be significantly different (Sign test; *p* = 0.0066).

The seasonal abundance observed in cattle sheds appeared to be noticeably different from that of houses and vegetation. The largest peaks in mean *P*. *argentipes* per trap-night by trap placement by trapping period were recorded during collection period-11 (June) in houses (110.4) and cattle enclosures (93.2) in Muzaffarpur and Saran, respectively (Figs 4B and 5B). In Muzaffarpur and Saran, significant differences were determined between cattle enclosures and houses (Sign test; *p* = 0.0066, *p* = 0.0015) and between cattle enclosures and vegetation (Sign test; *p*<0.0001, *p* = 0.0015). In Muzaffarpur and Saran, no significant differences were detected between houses and vegetation (Sign test*; p* = 0.8388).

### Comparing IRS-treated with untreated villages

**Fig 6:**
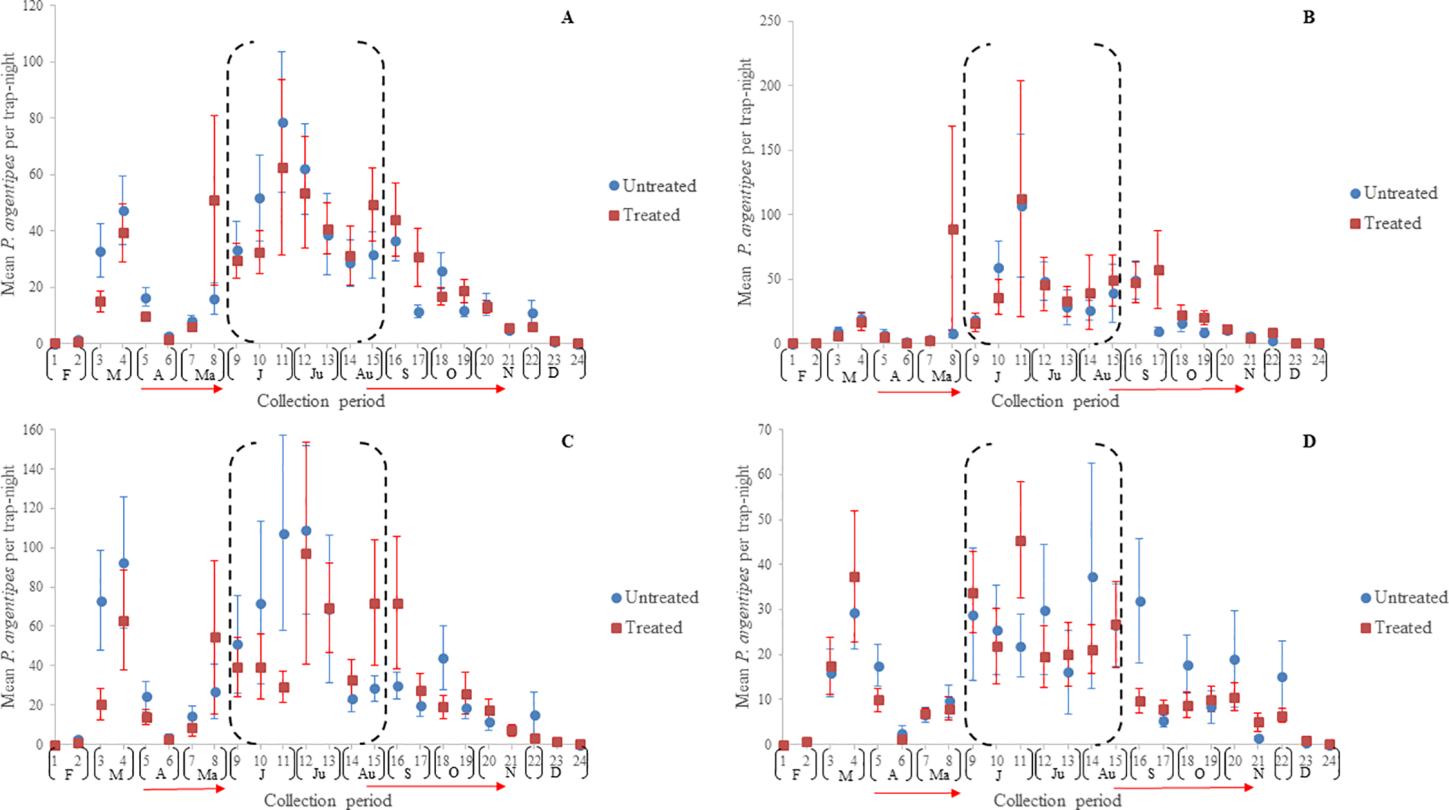
**Mean *P*. *argentipes* per trap-night in IRS-treated and untreated villages in Muzaffarpur in a) pooled locations, b) cattle enclosures, c) houses, and d) vegetation.** Vertical lines indicate the ±standard error (SE) and horizontal red arrows the range in 1^st^ and 2^nd^ round IRS spray dates. The black-dashed brackets (June-August) indicate the months of highest risk of human-vector exposure.

**Fig 7:**
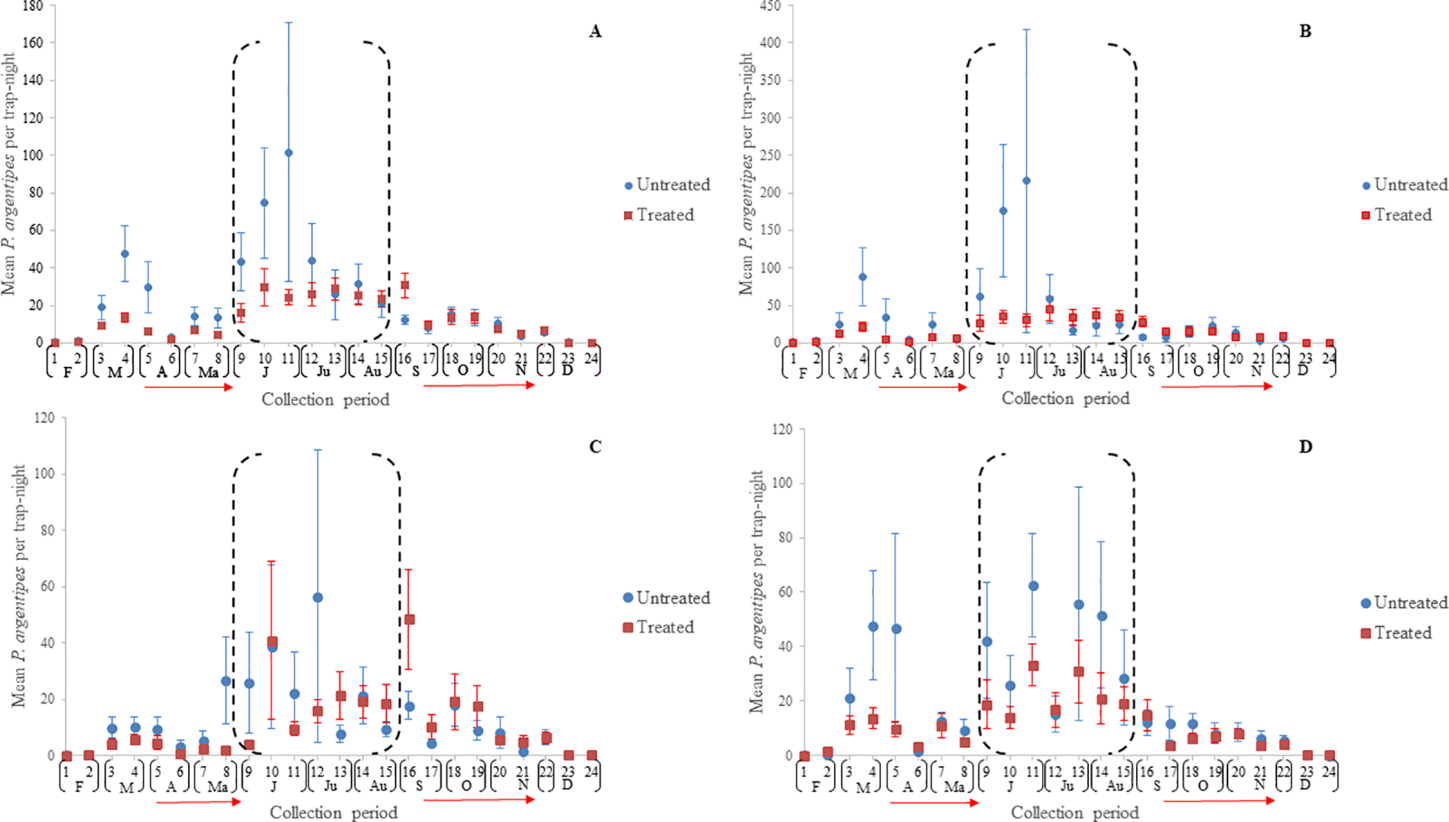
**Mean *P*. *argentipes* per trap-night in IRS-treated and untreated villages in Saran in a) pooled locations, b) cattle enclosures, c) houses, and d) vegetation.** Vertical lines indicate the ±standard error (SE) and horizontal red arrows the range in 1^st^ and 2^nd^ round IRS spray dates. The black-dashed brackets (June-August) indicate the months of highest risk of human-vector exposure.

The mean number of *P*. *argentipes* per trap-night per collection period in Muzaffarpur and Saran are indicated in Fig 6 and Fig 7, respectively. As noted, the first round of IRS applications was conducted between early April and late May to early June in both districts. Results of collections suggest that IRS-treatment failed to prevent the *P*. *argentipes* abundance in IRS-treated villages from increasing post-treatment June-August. Additionally, they indicate that if *P*. *argentipes* abundance was decreased in IRS-treated villages, the decrease was only temporary.

In Muzaffarpur, the relative *P*. *argentipes* abundance per trapping period in IRS-treated and untreated villages were similar (Fig 6A). The most notable difference occurred during collection period-11 (June) in houses where the mean *P*. *argentipes* per trap-night in IRS-treated villages was markedly lower (Fig 6C). However, the mean was comparable to the untreated villages during collection period-12.

In Saran, the mean number of *P*. *argentipes* in cattle enclosures suggested differences in *P*. *argentipes* abundance occurred in June (collection periods-9, 10, 11) (Fig 7B) but standard error was sizable because of outliers in one village (CI-210), particularly during collection period-11, and differences were less evident from collection period-12 onwards. Noticeable differences between *P*. *argentipes* abundance in IRS treated and untreated villages did not occur after the second round of IRS.

Nonparametric means of statistical analysis were used to estimate differences in *P*. *argentipes* abundance in IRS-treated and untreated villages because of the large standard errors reported. In total, of 40 cumulative evaluation periods (20 collection periods x 2 districts) and 120 trap placement evaluation periods (20 collection periods x 3 trap placements x 2 districts), significant differences were detected for 8 periods (20%) and 10 periods (~8%), respectively (S4 Table). Of 56 evaluation periods occurring June-August, statistical differences were detected in 5 periods (~9%), 4 occurring in June and 1 in July. Significant differences were most frequently detected during analysis of cumulative trap placements (*n* = 8) followed by houses (*n* = 5) cattle enclosures (*n* = 4) and vegetation (*n* = 1).

## Discussion

The results of this study 1) provide ecological data regarding *P*. *argentipes* monthly relative abundance, spatial distribution, and host preferance comparable to previous research; and 2) suggest relative *P*. *argentipes* abundance within IRS-treated and untreated villages do not differ significantly. To our knowledge, this is the most extensive entomology-based study, with insights into IRS-performance, conducted in Bihar, India.

Monthly *P*. *argentipes* relative abundance was comparable to results presented by [11], being highest in June, July and August and lowest in winter. *P*. *argentipes* spatial distribution was similar in that specimens were more frequently collected in cattle enclosures than other areas. Additionally, blood meal analysis suggested *P*. *argentipes* fed primarly on bovines and humans as reported by [17], but suggested a slight increase in the proportion taking full or partial blood meals from bovines (~60%: current; ~50%: previous).

The results of this study do not indicate that *P*. *argentipes* abundance in IRS-treated villages significantly differs from untreated villages in Muzaffarpur or Saran. Additionally, the application of IRS did not appear to prevent *P*. *argentipes* from increasing June-August. The lack of difference between IRS-treated and untreated villages is noteworthy, considering a recent mathematical, epidemiological model predicted a >67% reduction in *P*. *argentipes* abundance would be necessary to remove VL from a population [36]. [22] reported a 72% reduction in *P*. *argentipes* abundance resulting from IRS. However, these results are limited temporally, given that post-treatment collections were performed only twice in April, during which results of the current study and those of previous researchers [11,37] suggest sand fly abundance in Bihar declines naturally.

The efficacy of IRS is largely dependent on sand flies being endophilic [38] which should help explain why little difference was observed in *P*. *argentipes* abundance in vegetation. The presence of sand flies outdoors during this study and past studies [11,28,29] suggests a high percentage of the sand fly population may not come into contact with IRS-treated surfaces. One hundred and seventy three (173) *P*. *argentipes* collected in vegetation during the current study were confirmed to have taken a full or partial blood meal from humans. Considering the vaste majority of village households have members who sleep outdoors [8, 39], particularly during the warmer summer months, a proportion of *P*. *argentipes* may be able to feed and potentially transmit VL without ever entering a village dwelling. The increased likelihood of sleeping outdoors June-August, in conjunction with the greater vector density observed during this period in the current study, suggests villagers are placed at high risk of exposure to exophagic and exophilic *P*. *argentipes*.

For evaluation of IRS against exophilic, endophagic sand flies, the mortality rates of the insects entering sprayed households would be of interest. Researchers conducting a recent study in the Judean Desert of Jerusalem [40] observed that the number of sand flies captured in CO_2_ traps entering an experimental home (EH) with treated and untreated surfaces didn’t differ significantly, but noted that the mortality rate on deltamethrin-treated surfaces was higher when compared with untreated control (~69%, ~23%). While this would be useful information to obtain for the current study, the vaste number of insect species within CDC light traps, the process of collecting the large quantity of traps set per collection night (*n* = 144), and hot Indian temperatures made estimating sand fly mortality infeasible. However, the mean number of blood fed *P*. *argentipes* collected in CDC light traps positioned in homes in IRS-treated villages (*n* = 14.1) was comparable to untreated (*n* = 12), suggesting limited effect of IRS.

Although nonparametric statistical methods were used to account for outliers, the importance of these outliers and their potential implications in medical entomology and vector ecology should not be ignored. For this reason, we report the arithmetic means as well. A single house in one IRS-treated village (CI-58) in Muzaffarpur yielded 2,308 *P*. *argentipes* in a single trap (May 19), 33 days after being sprayed with alpha-cypermethrin. Another home located within the same village yielded 2,941 *P*. *argentipes* on June 30. In total, of six individual trap-nights which each yielded >1,000 *P*. *argentipes*, all were positioned within dwellings, and five were located in IRS-treated villages in Muzaffarpur. The sixth trap, which yielded 3,248 *P*. *argentipes*, was located in an untreated cattle enclosure in Saran (CI-210). It is unusual that dwellings in the latter village were not sprayed, given it was <1 km from an IRS-treated village (Fig 1), and interesting in that new cases were reported in 2016, leading to the village being sprayed on September 21 (S1 Table). The ability of some *Phlebotomus* spp. to fly distances >1–2 km [41–42], in addition to potential movement of VL-infected persons between villages, suggests that villages in close proximity to those with current VL cases should also be treated.

If vector density in Bihar has not decreased significantly in villages treated with IRS, it would suggest that the decline in reported VL cases [43] in Bihar could be related to other factors. Results of several studies indicate a repeating periodicity of VL in which peaks in cases occur every 10–20 years [44]. In the Goalpara District, Assam, five historical epidemic peaks were described by [45] occuring in 1885, 1897, 1913, 1925, and 1944. The three previous VL peaks in India have occured in 1978, 1992, and 2007 [46], a trend seen also in Bihar [47]. Researchers have suggested uncertainty as to whether the recent reduction in VL cases is a result of limited IRS efficacy or the natural VL cycle in south Asia [26], but given the abundance of *P*.*argentipes* in Bihar, the redundant periodicity of VL incidence, and the time since the most recent VL peak in Bihar, it is not unreasonable to suggest that the latter hypothesis may be correct. Other issues such as underreporting could be a factor, as many researchers believe the reported cases to be a gross underestimation of the true number [48–50]. Reported cases have been estimated to represent as low as 5–8% the total cases occuring in Bihar [51]. House to house surveys conducted in 14 villages in Bihar suggested only 12.3% of VL cases were officially reported [48].

Our results suggest that the current vector control strategy in Bihar may benefit from the integration of another vector control strategy aimed at targeting outdoor *P*. *argentipes*. One such vector control strategy to consider would be the treating of village vegetation with toxic sugar baits [52–53] an approach that exploits male and female *Phlebotomus* spp. sand flies tendency to feed on sugar from plants. Although this approach has not been pursued in Bihar, previous studies report a possible association between *P*. *argentipes* and Palmyra palm trees (*Borassus flabellifer*) [28–29] and banana plants (*Musa acuminata*) [29,54], suggesting these plant species may be candidates for sugar bait application.

The association of *P*. *argentipes* with cattle enclosures and bovine blood meals observed during this study suggests treating bovines systemically with approved drugs may also be a promising approach. Laboratory studies performed in Bihar confirm that endectocides administered systemically to lesser-bandicoot rats (*Bandicota bengalensis*) and roof rats (*Rattus rattus*) were highly efficacious against adult and larval *P*. *argentipes* [55]. A pen study during which fipronil was orally administered to cattle indicated near 100% mortality of adult female *P*. *argentipes* blood feeding on cattle 21 days after a single oral dose and 100% mortality of *P*. *argentipes* larvae feeding on feces collected from the same animals 21 days post-exposure [56]. The oviposition sites of *P*. *argentipes* are largely unknown, but when eggs, larvae, and/or pupae are found in Bihari villages, they are often found in proximity to cattle sheds [57–59], perhaps because the feces provide nutrition for developing larvae, which if confirmed would validate fipronil as a potential larvacide. We recommend the oviposition behavior of *P*. *argentipes* be explored at great length in future studies, as knowledge regarding larval habitat would greatly improve vector control schemes.

### Conclusion

We have collected abundant, contemporary ecological data from similar geographical locations in Bihar, which are suggested to produce more precise epidemiological models [60]. Our study is primarily an entomological field evaluation and in future studies the methods we implemented should be modified to include a baseline collection period and should be coupled with explicit epidemiological surveillance. Ecologically, our results suggest *P*. *argentipes* to 1) feed opportunistically on humans and bovines, 2) show a preference for cattle enclosures, and 3) be present outdoors in village vegetation. Because the majority of Bihari villagers sleep outdoors during periods when vector abundance is high, it is likely that many *P*. *argentipes* feed exophagically. This theory is supported by the lack of difference in *P*. *argentipes* abundance within IRS-treated and untreated villages, by previous field observations and blood meal analysis [11,17], and through observing *P*. *argentipes* infesting cattle during peak biting periods. Logically, IRS can only be efficacious in reducing endophagic vectors, which reinforces a need for supplemental vector control practices to reduce outdoor feeding populations. Because *P*. *argentipes* feeds heavily on bovines, endectocide-treated cattle may provide an appropriate means of reducing vectors unexposed to IRS-treated dwellings. By considering a complimentary form of vector control to better target exophagic, exophilic *P*. *argentipes* and by conducting explicit, frequent *P*. *argentipes* collection in combination with active and passive case detection, we could expand upon both integrated vector management and disease-vector surveilance, two of the main VL-reduction strategies discussed by the WHO [8]. As a result, we should be able to better protect outdoor-sleeping villagers and better estimate the sustainability of VL-reduction through program-initiated vector control.

## Supporting information

S1 Table List of IRS-treated and untreated villages and the dates of application for 24 villages in which sand flies were collected using CDC light traps from February 10-December 29. (XLSX)

S2 Table Mean maximum and minimum temperature and relative humidity and total rainfall recorded from February 1-December 31, 2016 in Patna, Bihar. (DOCX)

S3 Table Summary of the total number of sand flies collected from houses (H), cattle enclosures (CE), and peri-domestic vegetation (V) between February 10-December 29, 2016. (DOCX)

S4 Table Statistical differences in *P*. *argentipes* relative abundance. (DOCX)

S5 Table Sand flies collected, from February-December, 2016, from 24 villages in two districts in Bihar. (XLSX)

S1 Fig Microscopic images demonstrating the differences in appearance of a) male genitalia; and b) female spermatheca of *Phlebotomus argenitpes*, *P*. *papatasi*, and *Sergentomyia babu*, the three most common sand fly species collected in CDC light traps February 10-December 29, 2016.
(DOCX)

S2 Fig Comparison of spermatheca of *Grassomyia indica* and *Phlebotomus argentipes* females. (DOCX)
