# Supplementary material for: Bionomics of Phlebotomus argentipes in villages in Bihar, India with insights into efficacy of IRS-based control measures
Source: PLoS Negl Trop Dis. 2018 Jan 11;12(1):e0006168. doi: 10.1371/journal.pntd.0006168 (PMC5764230; doi:10.1371/journal.pntd.0006168)
Supplement: S2 Table — (DOCX) [file pntd.0006168.s002.docx]

| Month | Temperature (C) | | Humidity (%) | | Precipitation (mm) |
| --- | --- | --- | --- | --- | --- |
|  | Maximum | Minimum | Maximum | Minimum | Total |
| February | 28.7 | 14.0 | 89.1 | 28.4 | 0 |
| March | 34.1 | 18.7 | 77.3 | 21.7 | 4.06 |
| April | 40.6 | 23.5 | 59.6 | 16.9 | 0 |
| May | 36.1 | 23.6 | 82.9 | 37.9 | 76.72 |
| June | 36.9 | 26.5 | 85.5 | 44.7 | 50.03 |
| July | 33.0 | 25.4 | 95.2 | 64.9 | 202.94 |
| August | 34.1 | 25.3 | 93.2 | 59.0 | 87.88 |
| September | 32.4 | 24.0 | 97.7 | 69.1 | 181.62 |
| October | 33.2 | 21.9 | 96.3 | 49.5 | 50.04 |
| November | 29.7 | 15.6 | 94.0 | 39.2 | 0 |
| December | 21.6 | 11.8 | 99.5 | 62.3 | 0 |
