# Supplementary material for: Bionomics of Phlebotomus argentipes in villages in Bihar, India with insights into efficacy of IRS-based control measures
Source: PLoS Negl Trop Dis. 2018 Jan 11;12(1):e0006168. doi: 10.1371/journal.pntd.0006168 (PMC5764230; doi:10.1371/journal.pntd.0006168)
Supplement: S3 Table — (DOCX) [file pntd.0006168.s003.docx]

| District | Location | *Phlebotomus argentipes* | | | | | *Phlebotomus papatasi* | | | | | *Sergentomyia spp.* | | | | | F (*G. Indica*) | F (unknown) |
| --- | --- | --- | --- | --- | --- | --- | --- | --- | --- | --- | --- | --- | --- | --- | --- | --- | --- | --- |
|  |  | M | F (UF) | F (BF) | F (G) | Total | M | F (UF) | F (BF) | F (G) | Total | M | F (UF) | F (BF) | F (G) | Total |  |  |
| Muzaffarpur | H | 15052 | 10176 | 482 | 678 | 26388 | 73 | 85 | 1 | 2 | 161 | 1352 | 2031 | 31 | 94 | 3508 | 192 | 7 |
|  | CE | 21391 | 11729 | 634 | 920 | 34674 | 75 | 59 | 4 | 2 | 140 | 1211 | 1733 | 76 | 50 | 3070 | 304 | 8 |
|  | V | 9346 | 5531 | 176 | 401 | 15454 | 51 | 73 | 2 | 3 | 129 | 1677 | 2164 | 42 | 47 | 3930 | 609 | 7 |
|  | Total | 45789 | 27436 | 1292 | 1999 | 76516 | 199 | 217 | 7 | 7 | 430 | 4240 | 5928 | 149 | 191 | 10508 | 1105 | 22 |
|  | | | | | | | | | | | | | | | | | | |
| Saran | H | 7221 | 4137 | 160 | 434 | 11952 | 256 | 172 | 13 | 7 | 448 | 1100 | 1906 | 33 | 62 | 3101 | 278 | 0 |
|  | CE | 15252 | 7221 | 679 | 808 | 23960 | 130 | 130 | 12 | 6 | 278 | 1214 | 2220 | 104 | 57 | 3595 | 527 | 0 |
|  | V | 8642 | 5339 | 168 | 324 | 14473 | 155 | 156 | 6 | 4 | 321 | 2446 | 4551 | 37 | 42 | 7076 | 1314 | 4 |
|  | Total | 31115 | 16697 | 1007 | 1566 | 50385 | 541 | 458 | 31 | 17 | 1047 | 4760 | 8677 | 174 | 161 | 13772 | 2119 | 4 |
