# Supplementary material for: Bionomics of Phlebotomus argentipes in villages in Bihar, India with insights into efficacy of IRS-based control measures
Source: PLoS Negl Trop Dis. 2018 Jan 11;12(1):e0006168. doi: 10.1371/journal.pntd.0006168 (PMC5764230; doi:10.1371/journal.pntd.0006168)
Supplement: S4 Table — (DOCX) [file pntd.0006168.s004.docx]

| Month | Period** | Muzaffarpur | | | | Saran | | | |
| --- | --- | --- | --- | --- | --- | --- | --- | --- | --- |
|  |  | Cumulative | Cattle enclosures | Houses | Vegetation | Cumulative | Cattle enclosures | Houses | Vegetation |
| March | 3 | **0.0009***** | **0.0005***** | **0.0451***** | **-** | **-** | **-** | **-** | **-** |
|  | 4 | **-** | **-** | **0.0395***** | **-** | **-** | **-** | **-** | **-** |
| April | 5 | **0.0140***** | **-** | **-** | **-** | **0.0019***** | **-** | **0.0013***** | **-** |
|  | 6 | **-** | **-** | **-** | **-** | **-** | **-** | **-** | **-** |
| May | 7 | **0.0371***** | **0.0058***** | **-** | **-** | **-** | **-** | **-** | **-** |
|  | 8 | **-** | **-** | **-** | **-** | **-** | **-** | **-** | **-** |
| June* | 9 | **-** | **-** | **0.0160***** | **-** | **0.0176***** | **-** | **0.0110***** | **-** |
|  | 10 | **0.0192***** | **-** | **-** | **-** | **-** | **-** | **-** | **-** |
|  | 11 | **-** | **-** | **-** | **-** | **-** | **-** | **-** | **-** |
| July* | 12 | **-** | **-** | **-** | **-** | **-** | **-** | **-** | **-** |
|  | 13 | **0.0455***** | **-** | **-** | **-** | **-** | **-** | **-** | **-** |
| August* | 14 | **-** | **-** | **-** | **-** | **-** | **-** | **-** | **-** |
|  | 15 | **-** | **-** | **-** | **-** | **-** | **-** | **-** | **-** |
| September | 16 | **-** | **-** | **-** | **-** | **-** | **-** | **-** | **-** |
|  | 17 | **-** | **-** | **-** | **-** | **-** | **0.0138***** | **-** | **-** |
| October | 18 | **0.0077***** | **0.0169***** | **-** | **0.0448***** | **-** | **-** | **-** | **-** |
|  | 19 | **-** | **-** | **-** | **-** | **-** | **-** | **-** | **-** |
| November | 20 | **-** | **-** | **-** | **-** | **-** | **-** | **-** | **-** |
|  | 21 | **-** | **-** | **-** | **-** | **-** | **-** | **-** | **-** |
|  | 22 | **-** | **-** | **-** | **-** | **-** | **-** | **-** | **-** |

*The months of highest risk of human-vector exposure.

**The biweekly collection period

***Statistically significant differences in *P. argentipes* abundance in IRS-treated and untreated villages, determined by Wilcoxon rank sum test (*p* < 0.05).

-Indicates no statistically significant difference.
