# Supplementary figures and images for: Bionomics of Phlebotomus argentipes in villages in Bihar, India with insights into efficacy of IRS-based control measures
Source: PLoS Negl Trop Dis. 2018 Jan 11;12(1):e0006168. doi: 10.1371/journal.pntd.0006168 (PMC5764230; doi:10.1371/journal.pntd.0006168)

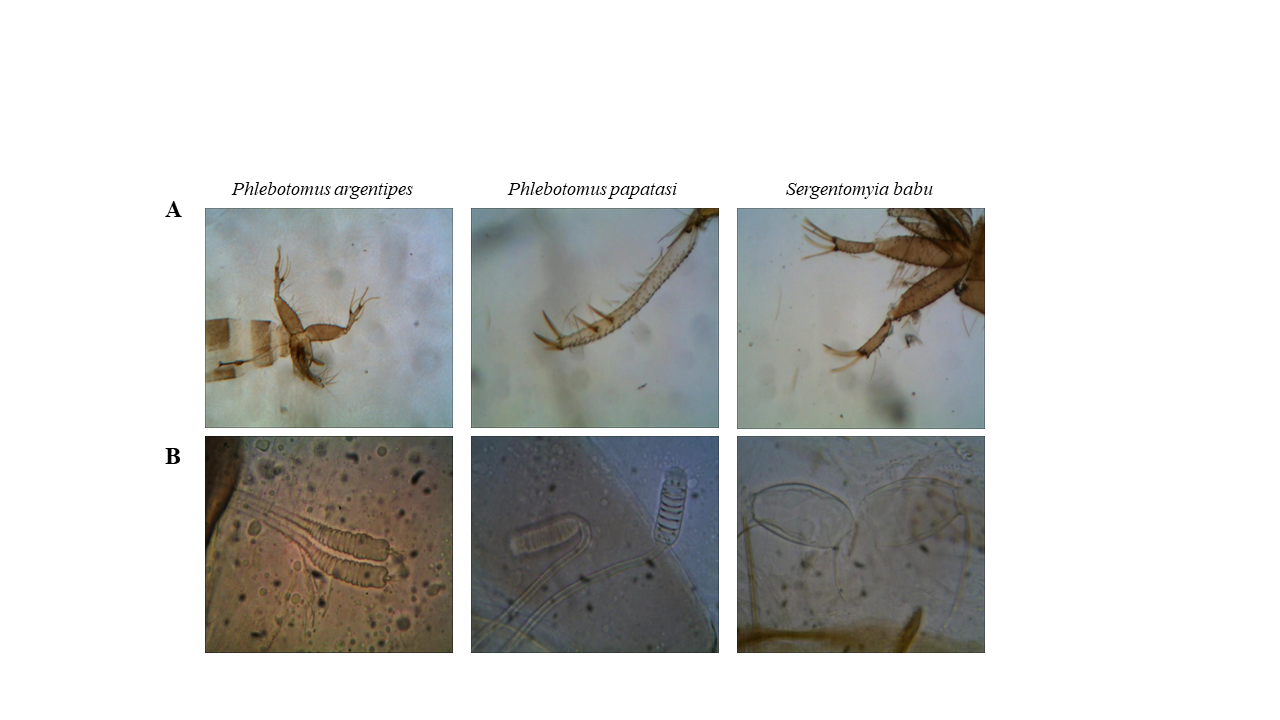

Supplement: S1 Fig — Microscopic images demonstrating the differences in appearance of a) male genitalia; and b) female spermatheca of Phlebotomus argenitpes, P. papatasi, and Sergentomyia babu, the three most common sand fly species collected in CDC light traps February 10-December 29, 2016. (DOCX) [file pntd.0006168.s006.docx]

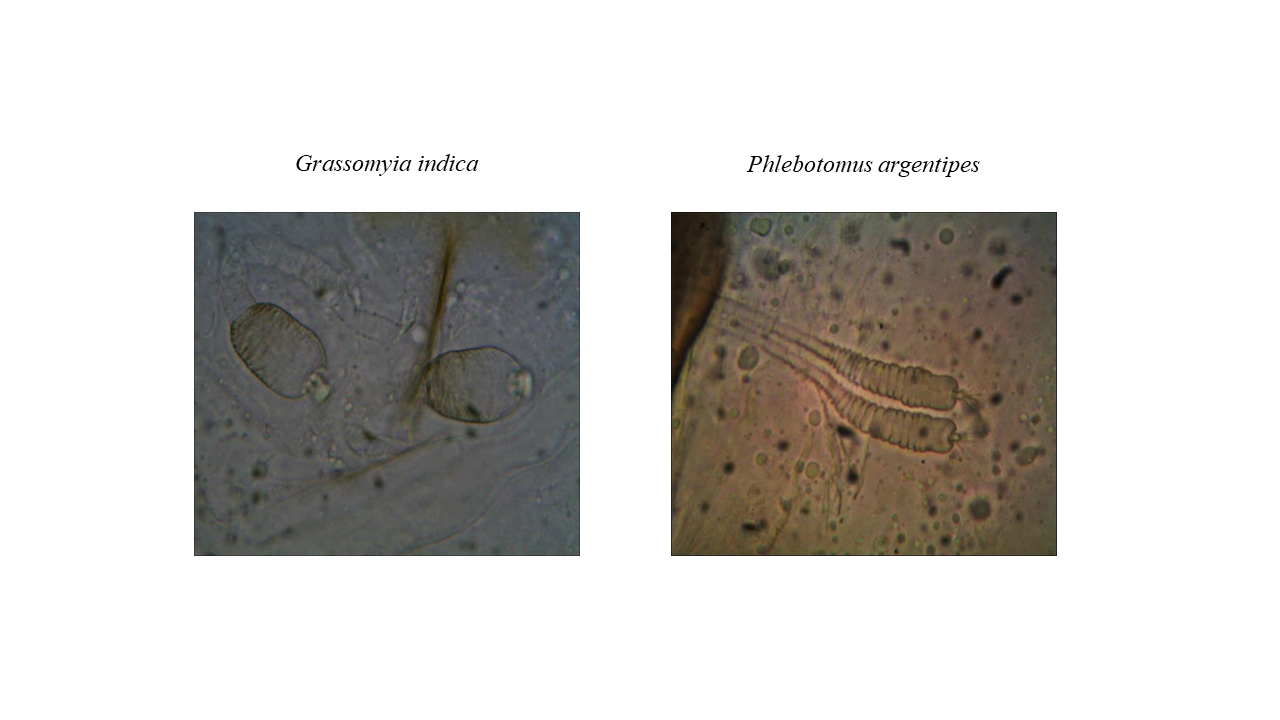

Supplement: S2 Fig — (DOCX) [file pntd.0006168.s007.docx]
